# Causal Modeling of Cancer-Stromal Communication Identifies PAPPA as a Novel Stroma-Secreted Factor Activating NFκB Signaling in Hepatocellular Carcinoma

**DOI:** 10.1371/journal.pcbi.1004293

**Published:** 2015-05-28

**Authors:** Julia C. Engelmann, Thomas Amann, Birgitta Ott-Rötzer, Margit Nützel, Yvonne Reinders, Jörg Reinders, Wolfgang E. Thasler, Theresa Kristl, Andreas Teufel, Christian G. Huber, Peter J. Oefner, Rainer Spang, Claus Hellerbrand

**Affiliations:** 1 Department of Statistical Bioinformatics, University of Regensburg, Regensburg, Germany; 2 Department of Internal Medicine I, University Hospital Regensburg, Regensburg, Germany; 3 Institute of Functional Genomics, University of Regensburg, Regensburg, Germany; 4 Biobank under the authority of Human Tissue and Cell Research (HTCR) and Center for Liver Cell Research, Department of General, Visceral, Transplantation, Vascular and Thoracic Surgery, Hospital of Ludwig-Maximilians-University of Munich, Munich, Germany; 5 Department of Molecular Biology, Division of Chemistry and Bioanalytics, University of Salzburg, Salzburg, Austria; Sahlgrenska academy at Göteborg university, SWEDEN

## Abstract

Inter-cellular communication with stromal cells is vital for cancer cells. Molecules involved in the communication are potential drug targets. To identify them systematically, we applied a systems level analysis that combined reverse network engineering with causal effect estimation. Using only observational transcriptome profiles we searched for paracrine factors sending messages from activated hepatic stellate cells (HSC) to hepatocellular carcinoma (HCC) cells. We condensed these messages to predict ten proteins that, acting in concert, cause the majority of the gene expression changes observed in HCC cells. Among the 10 paracrine factors were both known and unknown cancer promoting stromal factors, the former including Placental Growth Factor (PGF) and Periostin (POSTN), while Pregnancy-Associated Plasma Protein A (PAPPA) was among the latter. Further support for the predicted effect of PAPPA on HCC cells came from both *in vitro* studies that showed PAPPA to contribute to the activation of NFκB signaling, and clinical data, which linked higher expression levels of PAPPA to advanced stage HCC. In summary, this study demonstrates the potential of causal modeling in combination with a condensation step borrowed from gene set analysis [Model-based Gene Set Analysis (MGSA)] in the identification of stromal signaling molecules influencing the cancer phenotype.

## Author Summary

All living cells rely on communication with other cells to ensure their function and survival. Molecular signals are sent among cells of the same cell type and from cells of one cell type to another. In cancer, not only the cancer cells themselves are responsible for the malignancy, but also stromal (non-cancerous) cells and the molecular signals they send to cancer cells are important factors that determine the severity and outcome of the disease. Therefore, the identification of stromal signals and their influence on cancer cells is important for the development of novel treatment strategies. With a computational systems biology model of stroma-cancer cell communication, we have compiled a set of ten proteins secreted by stromal cells that shape the cancer phenotype. Most importantly, our causal analysis uncovered Pregnancy-Associated Plasma Protein A (PAPPA) as a novel paracrine inducer of the pro-tumorigenic NFκB signaling pathway. In liver cancer patients, higher levels of PAPPA protein indicate a more progressed tumor stage, confirming its clinical relevance.

## Introduction

Stromal tissue is a major component of solid tumors. It consists of extracellular matrix, connective tissue cells, inflammatory cells, and blood vessels. Stromal cells affect cancer development and progression by augmenting tumor cell proliferation, survival, motility and invasion [1,2,3]. Tumor and stromal cells can interact through both, direct cell-cell contact and secreted factors such as growth factors, cytokines, chemokines, and their cognate receptors [2,3].

Hepatocellular carcinoma (HCC) is one of the most prevalent and lethal malignant tumors worldwide. The major risk factor predisposing to HCC is hepatic cirrhosis. It arises through the activation of hepatic stellate cells (HSC), myofibroblast-like cells that are responsible for the excessive hepatic matrix deposition seen in chronically damaged livers [4,5]. Moreover, HSCs infiltrate the stroma of liver tumors localizing around tumor sinusoids, fibrous septa, and capsules [4,1]. Conditioned medium collected from activated HSCs induces growth, migration and invasion of HCC cells *in vitro* [6,7,8,9]. Furthermore, HSCs promote aggressive growth of HCC cells in experimental *in vivo* models [4,6,9,10] and their presence predicts poor clinical outcome in HCC patients [11]. These data indicate that HSCs affect HCCs. Yet, the molecular mechanisms of this crosstalk are largely unknown.

In functional assays, signaling pathways are analyzed through perturbation of the cellular systems. Unlike statistical associations in observational data, functional assays can directly distinguish between cause and effect. Their disadvantage is that they can be difficult to perform in high throughput.

Recently, Maathuis and colleagues introduced a novel method to extract causal information from observational gene expression data [12]. In their IDA algorithm they combine local reverse network engineering using the PC-algorithm [13] with causal effect estimation [14,15]. These virtual functional assays predict lists of genes that will change expression if the expression of a query gene was perturbed experimentally. The method was successfully applied to predict the expression profiles of yeast deletion strains from observational data of wild type yeast only [16].

Here, we adapt the IDA framework to the problem of identifying agents of inter-cellular communication. We combine a specific experimental design with tailored causal discovery and data integration algorithms. In brief, HSCs obtained from n = 15 human donors were cultivated to generate conditioned media for stimulation of the established HCC cell line Hep3B. Gene expression was then measured in both, HSCs as well as stimulated and un-stimulated HCC cells and a list of genes that change expression in HCCs upon stimulation was established. First, we aimed at identifying gene pairs (x, y) where the expression of gene x in HSCs affects the expression of gene y in HCC cells. Next, we searched for a small set of HSC expressed genes that, together accounted for the majority of stimulation sensitive genes in HCC cells. This yielded a set of 10 HSC genes predicted to jointly influence 120 of 227 HCC cell genes affected by supernatant stimulation.

## Results

### HSCs regulate oncogenic pathways in HCC cells

To study cell communication directed from stroma to cancer cells, we treated the HCC cell line Hep3B with 15 media conditioned by 24-hour cultivation with HSCs that had been isolated from different human donors. This design allows us to study the messages sent from HSCs to HCC cells independently from feedback messages that might be sent in the opposite direction from HCC cells to HSCs. The lack of feedback in this design is an indispensable prerequisite for our analytic approach.

**Fig 1:**
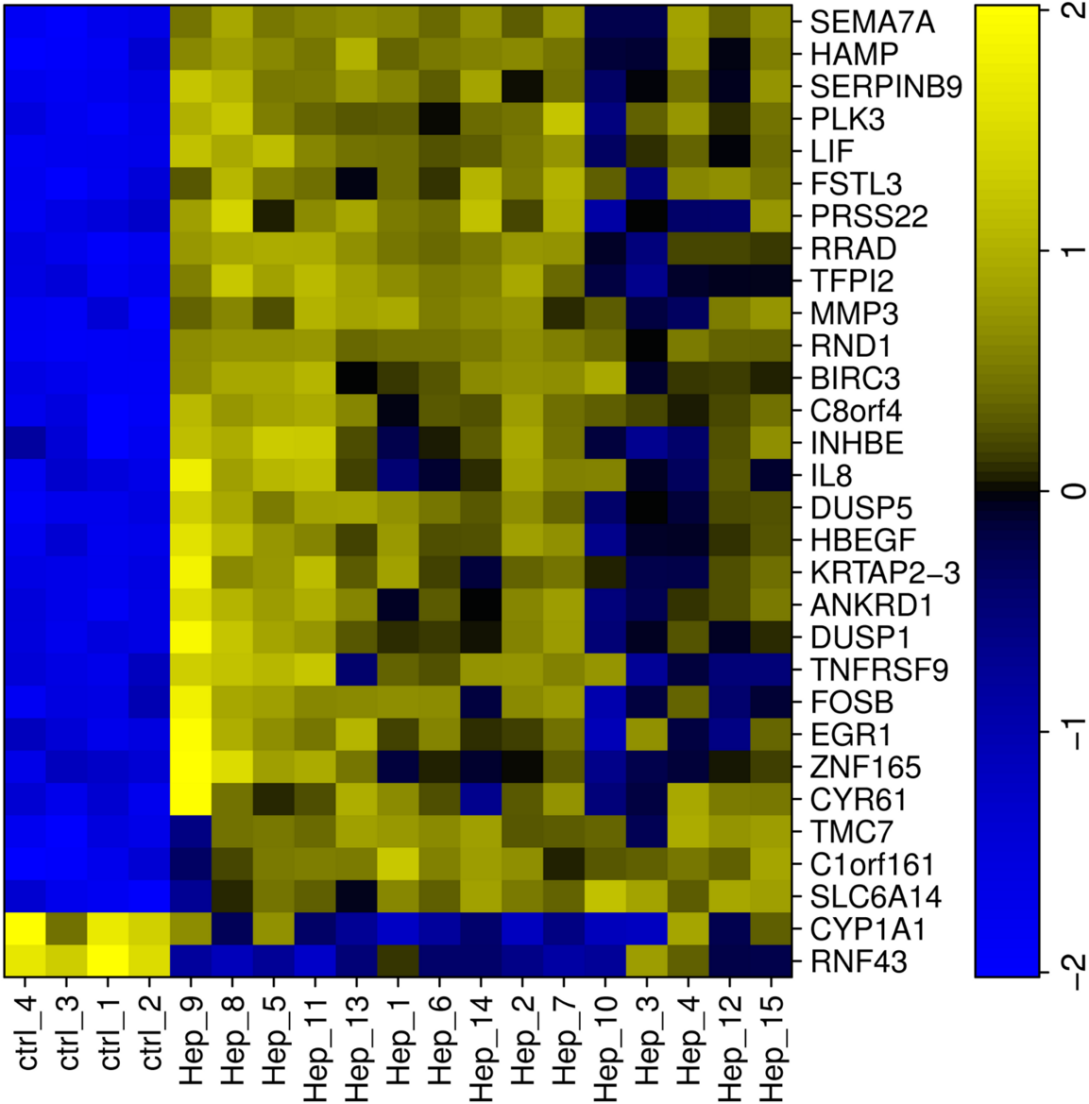
Differentially expressed genes with large variance across HCC samples. HCC cells were stimulated with conditioned media from HSCs from 15 different human donors (Hep_1-Hep_15) while control samples (ctrl1-4) were incubated with plain medium. Of the significant differentially expressed genes upon incubation with conditioned media, only the ones with large variation across HCC samples are shown (for details please see Material and Methods). Expression data was scaled to mean = 0 and standard deviation = 1, such that negative values (blue) indicate lower expression in the sample compared to the mean and positive values (yellow) higher expression in the sample compared to the mean.

Genome-wide gene expression was measured in both, donor HSCs and HCC cells stimulated with conditioned media (CM), yielding 15 pairs of gene expression profiles. The gene expression profiles of four un-stimulated HCC cell cultures served as controls. We identified a list of 227 genes with more than two-fold expression changes between stimulated and un-stimulated cells at an estimated false discovery rate (FDR) of 0.001. Interestingly, 30 (13.2%) of the 227 genes were among the top 200 genes with the highest variance in expression across the 15 stimulation assays (Fig 1). These genes reflect biological variation both across HSC donors and cancer cells stimulated by the HSCs. The genes that drive HSC induced neoplastic progression, including proliferation and migration in HCCs, are most likely among them [17]. In fact, testing for overrepresented Gene Ontology terms [18] pointed to several hallmarks of cancer: negative regulation of apoptosis (anti-apoptosis, q < 10^–4^), angiogenesis (q < 10^–4^), inflammation (cellular response to lipopolysaccharide, q < 10^–4^), positive regulation of cell migration (q < 10^–3^), and growth factor activity (transforming growth factor beta receptor signaling pathway, q < 10^–3^)(S1 Fig).

**Fig 2:**
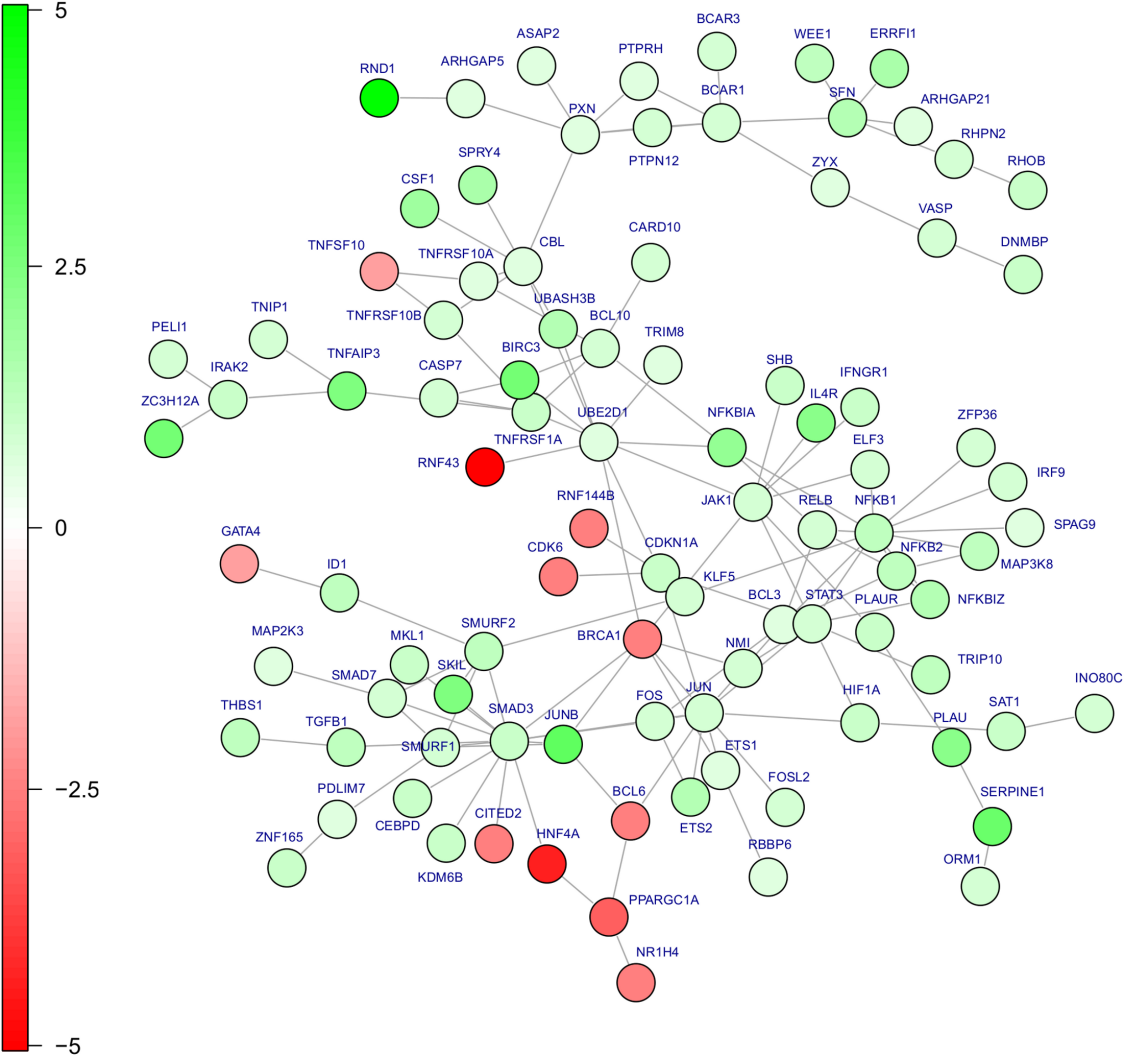
HCC protein network regulated by HSCs. HSC-induced changes in HCC gene expression were mapped on the BioGRID interactome of protein-protein and protein-gene interactions and the largest regulated sub-network was identified. Components of several oncogenic signaling pathways are regulated, NFκB pathway members, TGF-beta/SMAD3 and Map-kinases. Moreover, anti-apoptosis (BIRC3) and motility-related (RND1) genes can be found. Colors indicate logarithmic fold changes (base 2) of the genes upon conditioned media incubation. Red denotes repression; green induction of the gene after incubation with HSC conditioned media.

Next, we searched for indications which pathways might be regulated by stromal signals in HCC cells. The CM sensitive genes were mapped onto the BioGRID interactome of established protein-protein and protein-gene interactions [19] and the largest regulated subnetwork was identified by the BioNet algorithm [20]. The regulated network comprises several interacting oncogenic signaling pathways including TGF-beta/SMAD3, NFκB, JAK1 and MAP kinase signaling components (Fig 2). Another branch of the subnetwork can be attributed to anti-apoptotic signals with the highly induced *BIRC3* gene (ENSG00000023445) in its center. Amplification of the chromosomal region containing *BIRC3* exons is frequently found in HCC and associated with chemotherapy resistance, metastasis and poor prognosis [21]. The strongest induced gene, *RND1* (log2 fold change of 4.9; ENSG00000172602), a member of the Rho GTPase family [22], belongs to yet another branch of the subnetwork that comprises genes involved in regulating rearrangements of the actin cytoskeleton and, thus, changes in cell adhesion and motility in response to extracellular growth factors [23].

### Causal modeling identifies HSC secreted proteins affecting HCC cells

So far, we have only described the HSC-mediated changes in the HCC cell transcriptome. We have not yet identified the HSC secreted proteins that actually stimulate receptors or otherwise directly interact with HCCs. In a naïve analysis, we might find many genes in HSCs that correlate with some of the genes that are regulated in HCCs; however, most of them will not cause these changes.

**Fig 3:**
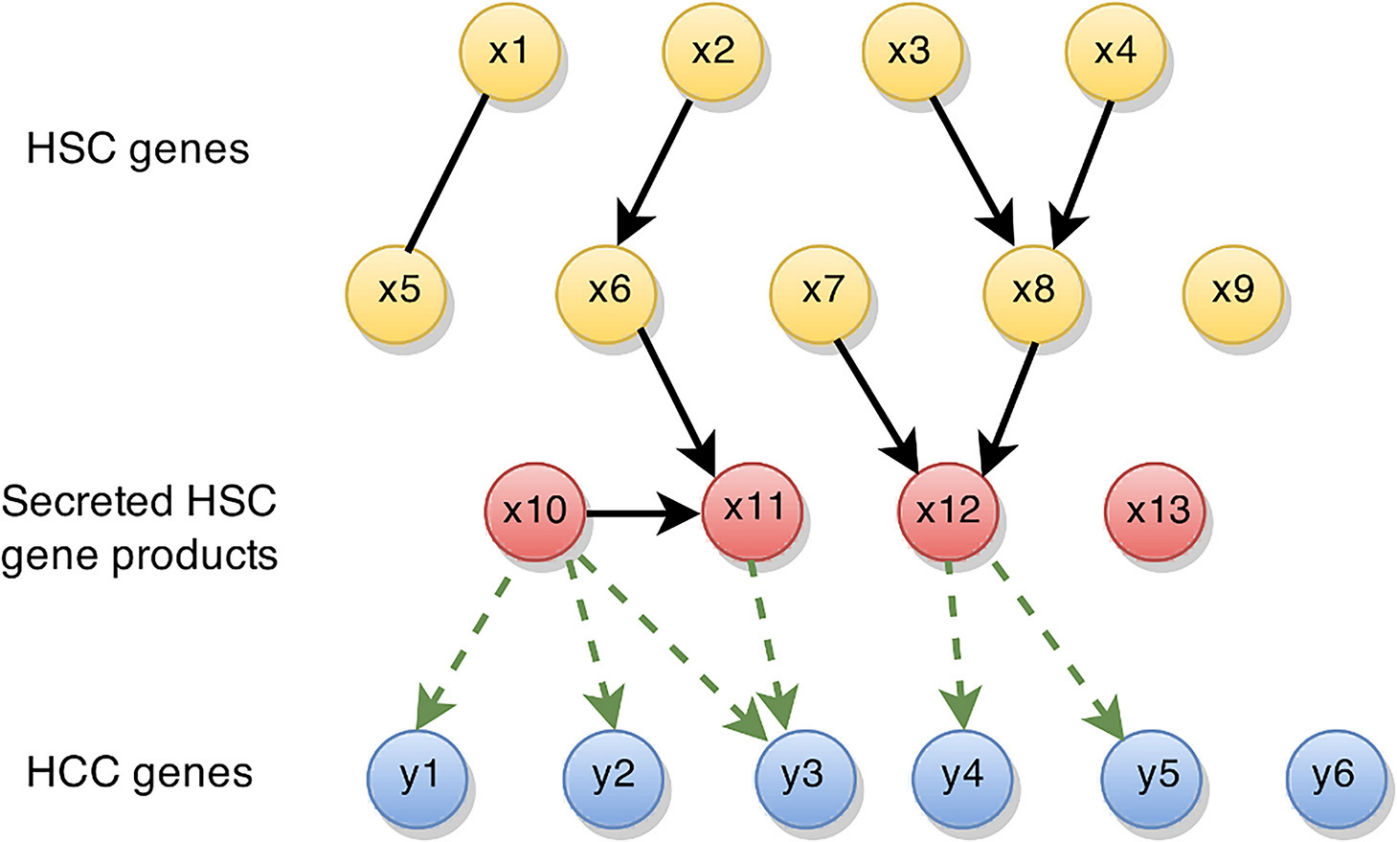
Scheme of the HSC-HCC network used in causal modeling. The network consists of three types of genes, cellular HSC genes (yellow), secreted HSC gene products (red) and HCC ‘target’ genes (blue). Individual genes are represented by nodes. Black arrows indicate dependencies among genes that were estimated from gene expression data. These can be directional, i.e. the expression level of a gene impacts the expression level of another downstream gene; or un-directed, i.e. the causal gene could not be inferred. Genes upstream of a particular gene are denoted as parents (e.g. x3 and x4 are parents of x8, and x3, x4, x7 and x8 are parents of x12). Secreted HSC gene products can be parents of other HSC genes. In contrast, HCC genes were excluded in network estimation because they cannot impact HSC genes in the chosen experimental setup. Green dashed arrows indicate estimated causal effects of secreted HSC genes on HCC cell genes. Causal effects that are stable across sub-sampling runs are reported, e.g. x10 has stable causal effects on y1, y2 and y3, whereas x13 has no stable effect on any HCC gene.

**Fig 4:**
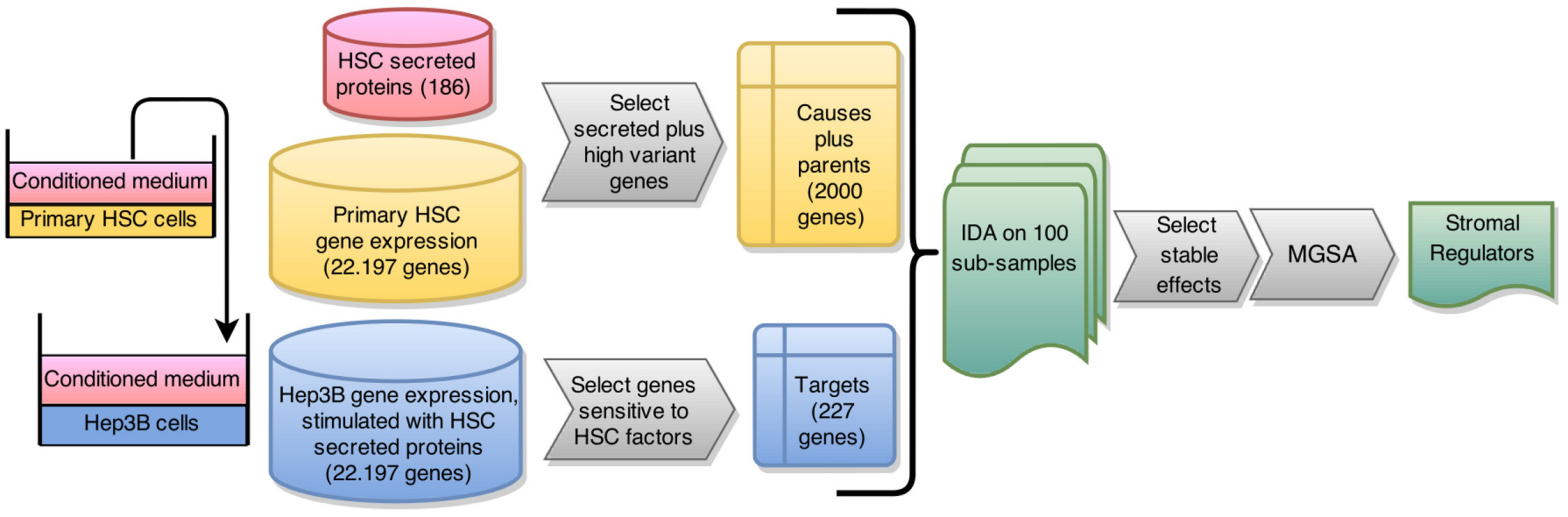
Overview of the experimental and computational approach to identify secreted factors of HSCs regulating HCC gene expression. Conditioned medium of primary human HSC (n = 15) was transferred onto human Hep3B HCC cells. Gene expression data of HSC and HCC cells were filtered to reduce the dimensionality of the data and to build cause-and-effect (target) matrices. These served as input for the IDA algorithm which estimates causal effects for each cause on each target gene. Causal effects that were stable across sub-sampling runs (i.e. that were stable with respect to small perturbations of the data) were retained and subjected to Model-based Gene Set Analysis (MGSA) to extract a sparse set of HSC genes influencing HCC cell gene expression.

In fact, if we counted the number of HCC genes a particular HSC gene correlates with (absolute Pearson correlation > 0.7), we would identify HSC-secreted POSTN (ENSG00000119655), PGF (ENSG00000119630), CSF1 (ENSG00000184371), NPC2 (ENSG119655) and FGF5 (ENSG00000138675). The top 10 list also includes HGF (ENSG00000019991) and is shown in S1 Table. Although this list points to potential stromal regulators, for some gene pairs correlation will be high due to a third factor that influences both of the correlated genes. To exclude the latter and to find true causal regulators instead, we use the “*in silico perturbation framework*” of the IDA algorithm [12] to filter for genes that are operative in stroma-to-tumor communication. Application of IDA comprises two steps. First, a partially directed network of regulatory interactions is constructed using the PC algorithm [13]. Second, causal effects are estimated from this network using Pearl’s Do-calculus [14]. To infer a potential effect of a stromal gene x on a cancer gene y, the Do-calculus needs the expression of y, x, and all genes x’ that generate spurious correlations between x and y (e.g. common regulators). Adjusting for the expression of the x’ (termed “parents of x”) differentiates between true causal effects and spurious correlations. If x does not have parents in the network (e.g. x10 in Fig 3), the estimated causal effect is identical to the correlation coefficient. However, if there are parents, causal effects are different from correlation coefficients. In these cases interpreting correlation coefficients is misleading. Since HSCs were never in contact with HCC cells, parent genes of x must be of HSC origin. Hence, it is sufficient to confine the reconstruction of a regulatory network to the HSC expression profiles only. An illustration of the HSC network is shown in Fig 3. To limit the computational burden resulting from genes that are not expressed in HSCs or that did not vary across HSCs from different donors, we only included the highest and most variably expressed genes (see Material and Methods) across the HSC samples in the analysis. The expression levels of HCC cell genes enter the model in the second step as y-genes, and the HSC network is used to derive causal effects of HSC on HCC genes (represented by green dashed arrows in Fig 3). For some genes, we have two expression values: one from the HSC sample that produced the CM, and one from the respective CM-stimulated HCC cell sample. For simplicity, we refer to these expression levels as the expression of the HSC gene and the HCC gene, respectively. For each of the 227 HSC-inducible HCC genes, we used IDA to screen for potential HSC genes that—when perturbed in expression—will have strong effects on the respective HCC gene. We limited our search for candidate HSC regulators to genes annotated as ‘secreted’, ‘extracellular’ or ‘intercellular’, but not ‘receptor’ by Gene Ontology and for which the gene product was detected in the conditioned media by HPLC/MS/MS. Gene products that are too small for detection, e.g. IGF1 (ENSG00000017427) and IGF2 (ENSG00000167244) were left in the analysis. This resulted in a final list of 186 HSC genes as candidate stromal regulators. The gene list with corresponding proteins can be found in S2 Table. Gene-pair-by-gene-pair, the HSC gene was *“virtually repressed”* by one standard unit and the expected change of the HCC gene was calculated. It is important to note that causal analysis will discover both direct and indirect effects of x on y, i.e. irrespective of potential mediators m, and discover effects of x and m if they are both secreted HSC genes. For example, in Fig 3, x10 has a causal effect on y3, although mediator node x11 also has a causal effect on y3. To be robust against small perturbations of the data, the *"virtual repression"* was run in a sub-sampling mode, repeating the experiment 100 times each on a different subset of the samples. Within each run, secreted HSC genes were ranked by the size of their estimated effects on the 227 target HCC genes. We kept causal effects only if they appeared in the top ranks across the majority of sub-sampling runs (see Material and Methods). This resulted in 96 HSC genes potentially regulating at least one of the 227 HCC genes. A flow-chart of our methodology is depicted in Fig 4.

### A small set of HSC secreted proteins can activate HCC cells in concert

Although all 186 HSC proteins have the potential to affect the expression of HCC genes, we postulate that a much smaller set of proteins is sufficient to activate HCCs. Thus we aimed at identifying a small set of HSC genes that jointly account for the wide spectrum of expression changes in HCC cells observed in response to stimulation with HSC-CMs. We have generated 227 lists of HSC regulators, one for each of the 227 CM sensitive HCC genes. Since many HSC genes were predicted to affect multiple HCC genes, these lists overlap. The lists can be reorganized by HSC genes instead of HCC genes. This resulted in 96 non-empty sets of HCC genes that are targeted by the same HSC gene.

Model based gene set analysis [24] (MGSA) is an algorithm that aims at partially covering an input list of genes with as little gene ontology categories as possible. It balances the coverage with the number of categories needed. We modified this algorithm in such a way that it covered the list of 227 CM sensitive HCC genes with the 96 sets of HSC targets. This strategy identified sparse lists of predicted targets that covered most of the observed targets. By definition, every list corresponded to one secreted HSC protein. This analysis brings HSC genes in competition to each other: an analysis based on frequencies (how many HCC genes does each HSC gene affect) discovers redundant HSC genes that target the same HCC genes. Our approach strives for a maximum coverage of the target genes with a minimum number of HSC secreted genes.

Both stability selection on the IDA algorithm and MGSA depend on the setting of a few parameters. Several studies have shown that hepatocellular growth factor (HGF) affects HCC cells [25], and is highly expressed in HSCs [25,26]. We exploited this knowledge and calibrated the parameters such that HGF appeared in the list of predicted HSC genes.

With these parameters, we identified 10 HSC secreted proteins. In addition to HGF the list included PGF, CXCL1, PAPPA, IGF2, IGFBP2, POSTN, NPC2, CTSB, and CSF1 (Table 1). With the exception of IGF2 all proteins were found in at least one of five CMs that were analyzed using LC/MS/MS. IGF2 is too small for successful detection [27]. Notably, the set of the most influential HSC regulators included several well-known tumor-promoting genes such as placental growth factor (PGF) [28], and the chemokine CXCL1, which promotes HCC angiogenesis and growth [29]. Periostin (POSTN) is a secreted cell adhesion protein whose expression levels are directly related to metastatic potential and poor prognosis of HCC [30]. High expression levels of the macrophage colony-stimulating factor 1 (CSF1) are another indicator of tumor progression and poor survival in HCC patients [31]. Over-expression of cathepsin B (CTSB), on the other hand, promotes HCC cell migration and invasion [32].

**Table 1:** Most influential stromal regulators.

symbol	ensembl gene ID	description	set size	probability
PGF	ENSG00000119630	placental growth factor	36	1
CXCL1	ENSG00000163739	chemokine (C-X-C motif) ligand 1	12	0.9951
POSTN	ENSG00000133110	periostin, osteoblast specific factor	25	0.9927
IGF2	ENSG00000167244	insulin-like growth factor 2	10	0.9864
PAPPA	ENSG00000182752	pregnancy-associated plasma protein A, pappalysin 1	9	0.9856
IGFBP2	ENSG00000115457	insulin-like growth factor binding protein 2, 36kDa	14	0.9843
CTSB	ENSG00000164733	cathepsin B	20	0.9501
NPC2	ENSG00000119655	Niemann-Pick disease, type C2	14	0.9300
HGF	ENSG00000019991	hepatocyte growth factor	16	0.8596
CSF1	ENSG00000184371	colony stimulating factor 1	8	0.7955

Subset of secreted HSC gene products which best describe the expression changes observed in conditioned HCC samples. symbol: gene symbol, ensembl gene ID: ensembl gene identifier, set size: number of HCC genes influenced by HSC gene product, probability: probability from MGSA that the target gene set is active (see Materials and Methods).

The role of Niemann-Pick Type C2 (NPC2) protein in cancer is just beginning to be understood. NPC2 regulates intracellular cholesterol homeostasis *via* direct binding with free cholesterol. Perturbations of cholesterol metabolism affect cancer progression [33]. Elevated serum levels of NPC2 have been observed in patients with lung cancer [34] and, more recently, HCC [35]. Modulation of cholesterol homeostasis by NPC2 also affects activation of mammalian target of rapamycin (mTOR) [36], a critical signaling cascade in several types of cancer including HCC [37].

Remarkably, we identified three genes of the insulin-like growth factor (IGF) axis. This signaling pathway regulates tumor progression in several types of tumors including HCC [38]. The key molecules in this pathway are the ligands IGF1 and IGF2, IGF-binding proteins (IGFBPs), and membrane-associated receptors (IGF-I receptor (IGF-IR), mannose-6-phosphate receptor/IGF-II receptor (IGF-IIR)). High expression levels of IGF2 are predictive of aggressive tumor growth and poor prognosis in HCC patients [39]. IGF2 binds to the receptor tyrosine kinases IGF1R (ENSG00000140443) and IGF2R (ENSG00000197081) on HCC cells and activates multiple intracellular signaling pathways, including the phosphatidylinositide-3′-kinase (PI3K)/Akt and MAP kinase signaling cascades [40]. IGFBPs bind to IGFs with higher affinity than IGF-receptors and, thereby, modulate local IGF concentrations and activities [40,41]. Unlike most IGFBP family members, which conduct antitumor activity, IGFBP2 promotes invasion, metastasis, and angiogenesis [41]. It is over-expressed in several tumor tissues including HCC [41,42].

The metalloprotease Pregnancy-Associated Plasma Protein A (PAPPA) is also a member of the IGF-axis. PAPPA is implicated in several biological functions [43], including the regulation of local IGF1 bioavailability through cleavage of IGFBPs [44]. Its expression in the liver under both, physiological and pathological conditions, including HCC development and progression, has not been elucidated yet. The few available studies on other tumor entities located PAPPA expression to cancer rather than stromal cells [45], and controversial roles of PAPPA regarding tumor progression have been reported in ovarian cancer [46]. Thus, we decided to focus our subsequent analysis on the role of PAPPA in HCC.

### Impact of parameter choice

In principle, parameters in our analysis could be set to different values and lead to different results. We evaluated the influence of gene pre-filtering and parameter settings in our analyses and found that the results were stable within the computationally feasible settings. Gene pre-filtering was necessary because network estimation is computationally very demanding with many genes. We evaluated our criteria for gene selection in a leave-one-out cross-validation and found that the selected genes are stable (secreted HSC genes: 95.1% identical with standard deviation (SD) 0.7%, intracellular HSC genes: 86.6% identical with SD 1.3%, HCC genes: 97.2% identical with SD 1.4%). S3 Table shows an aggregation of results when varying parameters in the causal analysis and demonstrates that these results are also stable. Among others, PAPPA is always within the top 10 stromal regulators.

### PAPPA activates NFκB signaling in HCC cell lines

**Fig 5:**
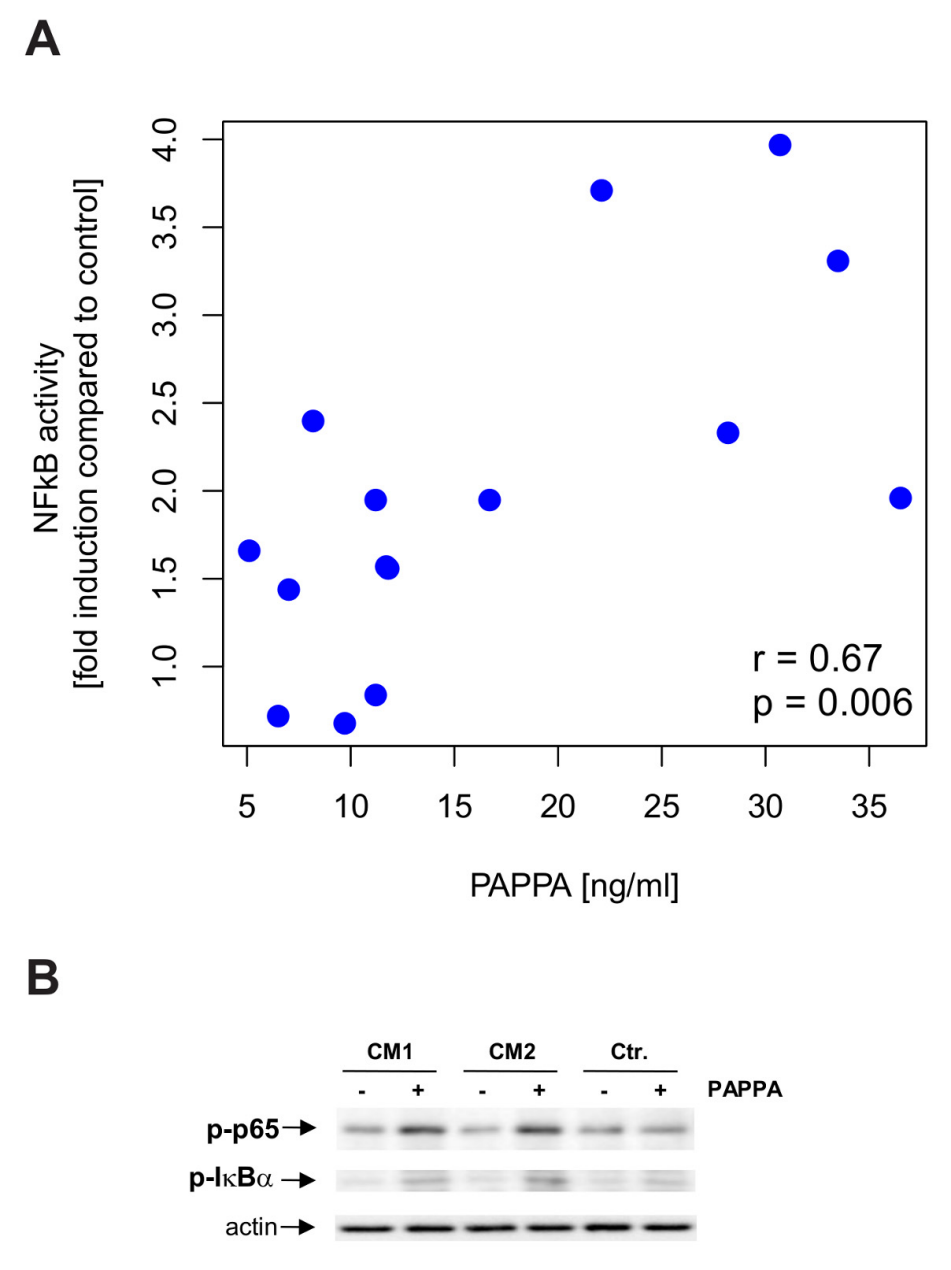
Correlation of HSC secreted PAPPA levels with NFκB activation in conditioned HCC. A. Correlation of HSC-CM induced NFκB activity in HCC cells (relative to NFκB activity in cells stimulated with control medium) with PAPPA levels in HSC-CM (n = 15). B. HCC cells were incubated with recombinant human PAPPA protein (PAPPA) either in CM from HCSs from 2 different human donors (CM1 and CM2) or control medium (ctr.). Furthermore, cells were stimulated with CM1, CM2 or control medium alone. After 4h stimulation, cellular extracts were analyzed with Western blot analysis for phosphorylated p65 and IkB-alpha. Analysis of actin expression demonstrated equal loading.

The list of CM sensitive HCC genes includes various members of the NFκB pathway (Fig 2 **;** NFKB1 (ENSG00000109320), NFKB2 (ENSG00000077150), NFKBIZ (ENSG0000014480), NFKBIA (ENSG00000100906), RELB (ENSG00000104856)) and targets of the NFκB pathway previously collected by Compagno et al [47], such as BIRC3, EGR1 (ENSG00000120738), ICAM1 (ENSG00000090339), IL8 (ENSG00000169429), MAP3K8 (ENSG00000107968)**.** Several of these genes were predicted to be targets of HSC secreted PAPPA by our causal analysis (ICAM1, MAP3K8, NFKBIA, see S4 Table for the full list). Also the other predicted target genes are known to be regulated by the transcription factor NFκB or to affect this signal transduction pathway [48,49,50,51,52,53]. To test whether PAPPA might be indeed responsible for activation and auto-regulation of the NFκB pathway, we assessed NFκB activity in stimulated HCC cells and observed a striking correlation of PAPPA levels in conditioned medium (CM) from the 15 different HSCs with NFκB activity induced in HCC cells upon incubation with these different CMs (Fig 5A). To verify a causal effect of PAPPA on NFκB activity in HCC, we stimulated Hep3B HCC cells with recombinant human PAPPA protein (rPAPPA). We applied rPAPPA (25 ng/ml) either alone or in CM of HSCs from two different donors containing endogenous PAPPA levels of 4.8 ng/ml and 6.2 ng/ml, respectively. In control medium, rPAPPA did not significantly affect IkB-α- and p65-phosphorylation, while together with CM both IkB- α- and p65-phosphorylation were higher than in CM-stimulated cells (Fig 5B).

### PAPPA is expressed in human HSCs but not in HCC cells

**Fig 6:**
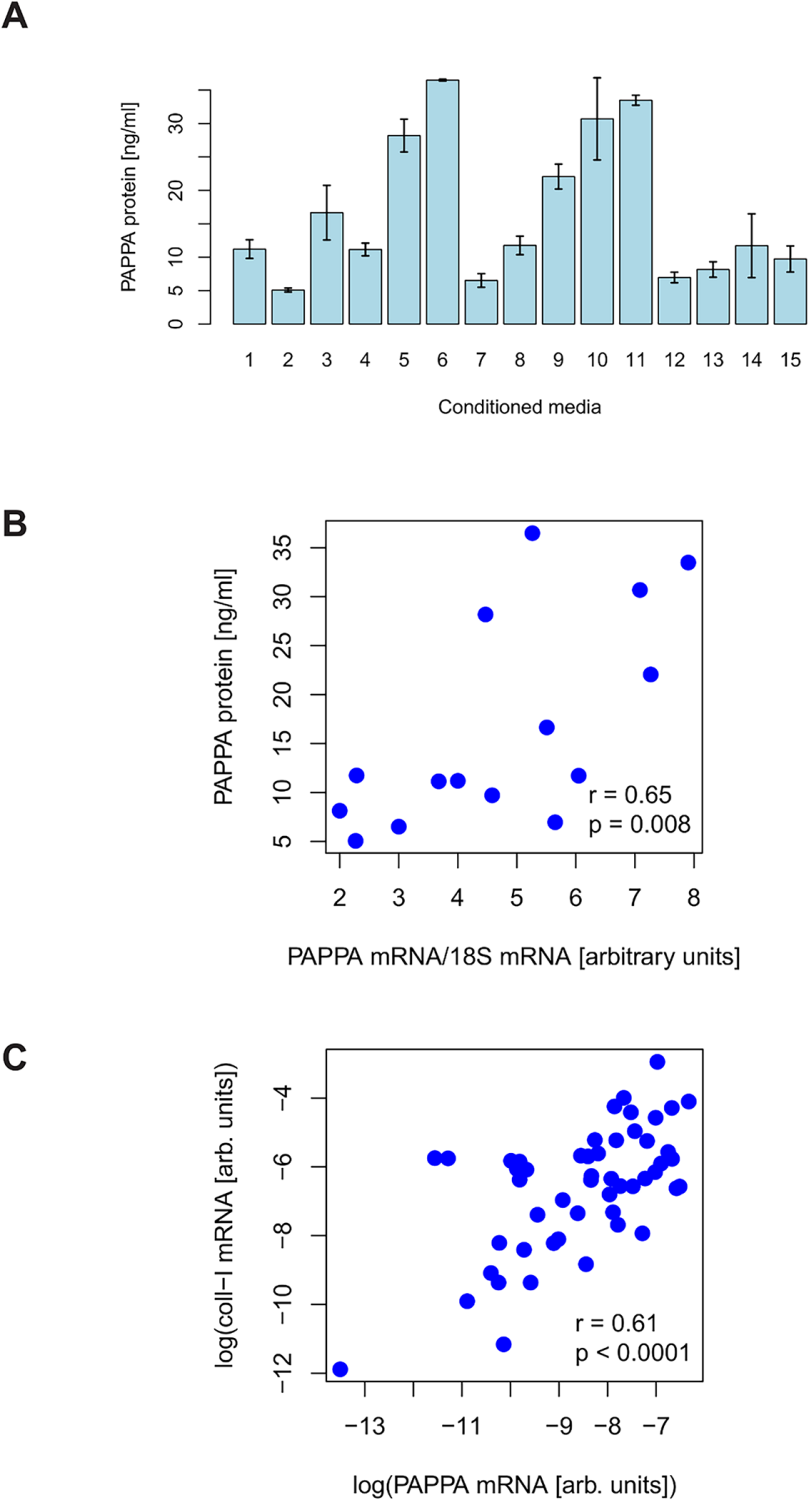
PAPPA expression in HSCs and HCC tissues. PAPPA protein levels in conditioned media, correlation of protein and mRNA levels, and correlation with collagen. A. PAPPA levels in conditioned media of HSCs from 15 different human donors. B. Correlation of PAPPA protein levels and mRNA levels in HSCs from 15 different human donors. C. Correlation of PAPPA and collagen I (COL1A1) mRNA expression in 51 human HCC tissues.

Quantitative real time PCR analysis showed strong PAPPA mRNA expression in HSCs whereas no expression was detectable in 4 different human HCC cell lines including Hep3B (S2 Fig). Concordantly, PAPPA protein levels ranged from approximately 5 to 35 ng/ml in supernatants of HSC cultures, while no PAPPA protein was detectable in the supernatants of the 4 different HCC cell lines (Figs 6A and S3). In the 15 different HSCs, we observed a significant correlation between mRNA and protein levels of PAPPA (Fig 6B), indicating that secreted PAPPA levels are regulated at the transcriptional level. Next, we assessed PAPPA gene expression in HCC specimens from 52 patients and found a significant correlation with collagen type I (COL1A1; ENSG0000010882) mRNA expression (Fig 6C). This finding could be confirmed in the HCC cohort of The Cancer Genome Atlas (TCGA, http://cancergenome.nih.gov) (S4 Fig). HSCs infiltrate and form the HCC stroma and collagen type I is specifically expressed by HSCs in HCC tissue [4,54,5]. Together, these findings indicate that HSCs are the major source of PAPPA in HCC.

### PAPPA expression correlates with HCC progression *in vivo*

**Fig 7:**
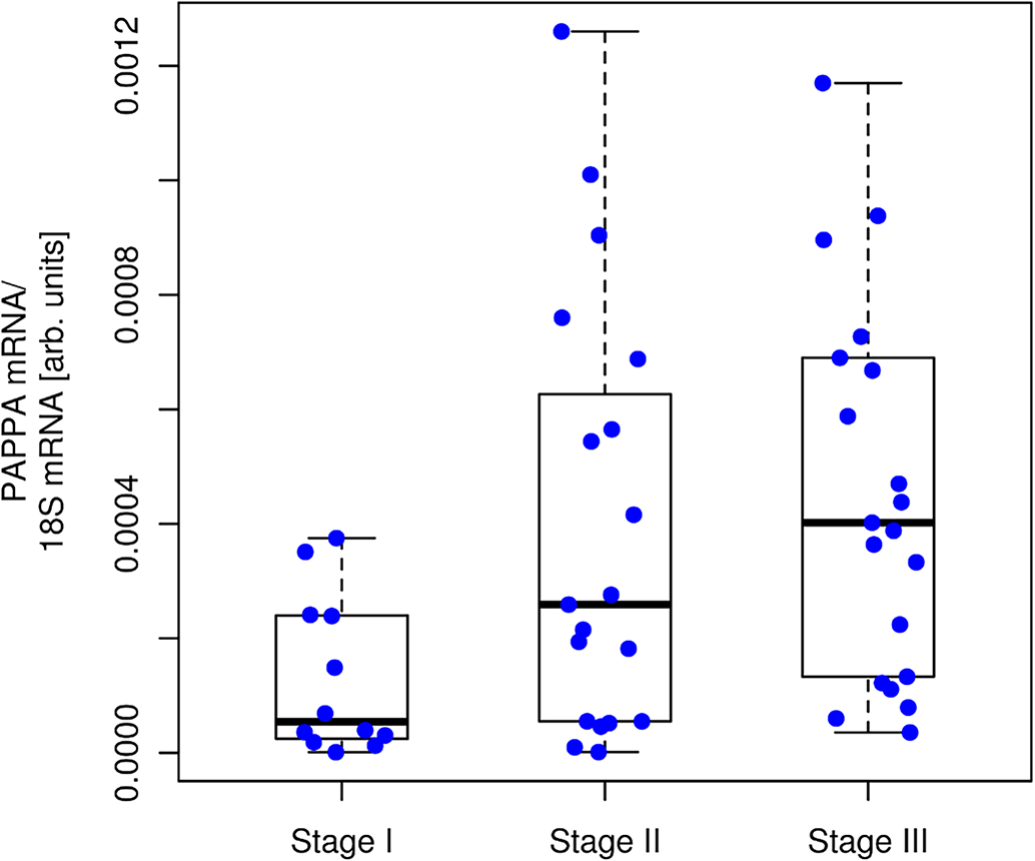
PAPPA expression in human HCC tissues of different tumor stages. PAPPA mRNA expression levels in human HCC tissues (n = 52) of tumor stages I (n = 12), II (n = 19) and III (n = 21). One-way ANOVA shows a significant effect (p = 0.008) of tumor stage on PAPPA mRNA expression level.

Histological staging of HCC is a prognostic factor of patient survival in HCC [54,55,56]. We found that PAPPA expression in human HCC specimens (n = 52) was significantly lower (p = 0.008, one-way ANOVA) in patients with low histological staging (stage I; n = 12) compared to patients with stage II (n = 19) and stage III (n = 21) disease (Fig 7). In an independent data set, the HCC cohort of TCGA, PAPPA expression was also significantly lower in stage I patients (n = 104) compared to stage II (n = 56) and stage III (n = 39) in a one-way ANOVA (p = 0.0126) (S5 Fig). Together, these findings indicate the clinical relevance of HSC secreted PAPPA for HCC progression.

## Discussion

Introductory statistical text books stress the difference between association and causation. For example, correlation between the expression levels of two genes does not imply that one gene regulates the other. They can as well be co-regulated by a third gene. The gold standard to infer causalities is experimental intervention. If a knock-down of the first gene changes the expression of the second, there is a functional relation between the two. In fact, the rationale of functional genetics is to understand the cell by breaking it. Functional assays that perturb biological networks experimentally shed light on cellular mechanisms.

Causal inference from observational data is a more advanced statistical discipline [13,14] that only recently found its way into bioinformatics and systems biology after a statistical breakthrough paper by Maathuis et al. (2009) [12]. To date it has been used for the analysis of yeast deletion strains [16], to predict genes regulating flowering time in *Arabidopsis thaliana* [57], and for the prediction of miRNA targets [58]. Here, we add another biological application to this list: The identification of secreted proteins that drive inter-cellular communication in human cancer.

State of the art statistical methodology does not allow for feedback mechanisms between the regulator and its target. This is an assumption that nature does not meet in many cases. In a tumor it is most likely that the communication between stromal and tumor cells is mutual. In our experimental setting however, feedback is blocked. Stromal and cancer cells grow in separate cultures. The stromal cells "talk" to the cancer cells *via* the CMs but there is no "reply".

Clearly, this does not give us a full picture of cellular communication; feedback mechanisms are blocked and so are signals mediated by cell-cell contacts. But it is this focus on unidirectional paracrine signaling that allows us to use causal modeling. The experimental design is tailored to the capabilities of the predictive model. In spite of these limitations our application to HCC demonstrates that the method can generate novel and potentially clinical relevant insights into the mechanisms of stroma-tumor communication. We unmasked PAPPA as a novel stroma secreted factor impacting the tumor phenotype.

Notably, our 10 HSC secreted regulators did not only include PAPPA but two more genes of the IGF-axis. The IGF-axis is one of the molecular networks involved in the formation, progression and metastatic spread of many cancer types, including HCC. IGF2 and IGFBP2 are known to critically affect HCC development and progression. Still, most studies focused on autocrine effects of these two secreted proteins in cancer cells, while our data suggest a paracrine effect whereby HSC derived IGF2 and IGFBP2 influence IGF-signaling in HCC cells.

The expression and function of PAPPA in normal and diseased liver were not known thus far. To date, PAPPA has been mainly used as a biomarker in prenatal screening for Down's syndrome [43]. More recently, PAPPA has been identified as a regulator of the bioavailability of IGFs through the cleavage of IGF binding proteins [43,59]. It has been suggested to exert a protumorigenic role in breast cancer, lung cancer, and malignant pleural mesothelioma [59]. In contrast, breast cancer cells have been reported to become more invasive after down-regulation of PAPPA [60]. Controversial roles of PAPPA have also been reported in ovarian cancer, with most ovarian cancer cell lines and primary tumors showing partial or complete loss of PAPPA expression [45]. Furthermore, PAPPA expression was shown to be consistently high in normal ovarian specimens, while it was suppressed by SV40 large T antigen [61].

In HCC, our data suggest PAPPA as a protumorigenic factor. We found significantly higher PAPPA expression levels in advanced stage tumors. On the mechanistic side, we found that PAPPA induces NFκB-activity in HCC cells. We observed a significant correlation between PAPPA levels in different conditioned media of HSCs and corresponding effects on NFκB activation in HCC cells *in vitro*.

Interestingly, studies in ovarian, breast and lung cancer as well as malignant pleural mesothelioma revealed the cancer rather than the stromal cells as the cellular source of PAPPA. Here, in contrast, PAPPA expression was only detected in HSCs, but not in HCC cells. This makes PAPPA a promising therapeutic target in HCC, as tumor stromal cells are genetically more stable than cancer cells, which renders them less likely to evade therapy. Moreover, it has to be considered that the IGF-axis also plays a critical role in HSC activation and fibrosis [62]. Although the function of PAPPA in HSCs is unknown, it may be speculated that PAPPA inhibition may suppress the fibrogenic phenotype of HSCs. Since HCC mostly develops in cirrhotic liver tissue [1,4], inhibition of PAPPA could not only affect HCC cells but also prevent the formation of a protumorigenic soil for cancer cells.

Due to its central role in cancer progression, a variety of reagents have been developed to modulate IGF signaling including neutralizing antibodies against IGFs and IGF-receptors as well as associated receptor kinase inhibitors in aim for cancer treatment [63]. The structural similarities of the insulin and IGF-IRs complicate the development of specific agents that block IGF-IR signaling without affecting insulin signaling. This is particularly true with regards to treatment of liver cancer due to the central role of the liver in glucose metabolism and homeostasis. In contrast to the persistent and versatile physiological functions of other components of the IGF1 axis, PAPPA could not be detected in normal human liver and primary human hepatocytes (S6 Fig). Therefore, PAPPA appears as a better therapeutic target for HCC with more tumor specificity and less risks of side effects as compared to other IGF1 axis components. Actually, genetic deletion of PAPPA extended lifespan of mice [59,64].

In conclusion, we have shown for the first time that causal modeling can be used to identify stromal signaling molecules that influence the cancer phenotype. Application of our modeling strategy unmasked PAPPA as a novel paracrine factor that shapes the tumor phenotype via activating the NFκB pathway.

## Materials and Methods

### Ethics statement

Human liver tissues were obtained and experimental procedures were performed according to the guidelines of the charitable state controlled foundation HTCR (Human Tissue and Cell Research), with the informed patients’ consents, and approval by the local ethics committee of the Ludwig-Maximilians University of Munich (reference number 025–12). All experiments involving human tissues and cells have been carried out in accordance with The Code of Ethics of the World Medical Association (Declaration of Helsinki).

### Cells and cell culture models

The human HCC cell lines Hep3B (American Type Culture Collection (ATCC) number HB-8064), HepG2 (ATCC; HB-8065), PLC (ATCC; CRL-8024) and Huh-7 (Japan Collection of Research Bioresources (JCR) number B0403) were cultured as described [10,65].

Primary human hepatic stellate cells (HSCs) were isolated from 15 different human donors as described [10,66,67]. The isolation procedure and cell culture on uncoated tissue culture dishes led to the activation of HSCs as described [66,67]. For collection of conditioned medium (CM), HSCs were seeded into T75 flasks (2 × 10^6^ cells). One day after seeding cells were washed twice with serum-free DMEM, and then incubated for another 24 h with serum-free DMEM (15 mL/T75). CM was centrifuged at 6,000 x g to remove cell debris, sterile filtered (0.45 μm pore size membrane filter), and stored in aliquots at −80°C until use. Serum-free DMEM incubated for 24 h in cell culture flasks without cells served as the control.

For stimulation with HSC conditioned media, HCC cells were seeded into T25 flask (10^6^ cells). One day after seeding, cells were washed with serum-free DMEM, and then incubated for another 12 h with serum-free DMEM. Subsequently, the medium was changed and cells were incubated with 3 mL of HSC-CM or control medium (serum-free DMEM) for 4 h.

For individual experiments, CM was preincubated with recombinant PAPPA (R&D Systems, Wiesbaden, Germany).

HCC tissues were obtained from HCC patients undergoing surgical resection. Tissue samples were immediately snap-frozen and stored at -80°C until analysis.

### RNA extraction and gene expression analysis

Isolation of total cellular RNA from cultured cells and tissues and reverse transcription were performed as described [10,65]. 300 ng of RNA were hybridized to Affymetrix Human Gene ST 1.0 arrays following the standard Affymetrix protocol (Affymetrix, High Wycombe, UK). Hybridization and scanning were performed at an Affymetrix Service Provider and Core Facility, “KFB—Center of Excellence for Fluorescent Bioanalytics” (Regensburg, Germany; www.kfb-regensburg.de).

Quantitative real-time-PCR was performed applying LightCycler technology (Roche, Mannheim, Germany) and the following pairs of primers: human PAPPA (forward: 5'-AGC CAG CAG CAT CCC AGG TGT-3'; reverse: 5'-CGC CCG GAG CCA AAA AGT GGT)-3' and human collagen type I (forward: 5'- CGG CTC CTG CTC CTC TT -3'; reverse: 5'-GGG GCA GTT CTT GGT CTC -3'). Amplification of cDNA derived from 18s rRNA (forward: 5'-TCT GTG ATG CCC TTA GAT GTC C-3'; reverse: 5'-CCA TCC AAT CGG TAG TAG CG-3') was used for normalization.

### Western blot analysis

Protein extraction and western blotting analysis were performed as described [65] applying antibodies against phospho-NF-κB p65 ((Ser536) rabbit mAb #3033) and phospho-IκBα ((Ser32); rabbit mAb #2859) both from Cell Signaling Technology (Danvers, MA, USA; all diluted 1:1,000). Furthermore, an antibody against actin (MAB1501 from Merck Millipore, Billerica, MA, USA; 1:1,000) was applied.

### Quantification of activated nuclear NFkB concentration

Activated NF-κB was quantified in nuclear extracts with the ELISA based kit TransAm from Active Motif (Rixensart, Belgium) according to the manufacturer's instructions, as described [66].

### Pre-processing of microarray data

Normalization of raw intensity values from CEL files was performed using variance stabilization (VSN) [68]. Median polish and a custom chip description file based on ensembl gene identifiers [69] were used to summarize individual probes to obtain an expression level per gene. Raw intensities and normalized gene expression data are available publicly at the NCBI Gene Expression Omnibus (GEO, http://www.ncbi.nlm.nih.gov/geo/) under accession GSE62455. Differential gene expression between Hep3B cells treated with different CMs and untreated Hep3B controls was estimated using limma [70]. All analyses were performed within the statistical programming environment R.

### Gene Set Analysis, network analysis

Gene Set Analysis (GSA) was performed using hypergeometric tests implemented in the Bioconductor package HTSanalyzeR [71]. Genes meeting the FDR threshold of 0.001 and an absolute log2 fold change larger than one were selected for testing significant enrichment of Gene Ontology (GO) terms within the Biological Process (BP) branch. The Bioconductor package BioNet [20] was used to find the highest-scoring sub-network within the differentially expressed genes with FDR < 0.001 and an absolute log2 fold change larger than 0.7.

### Proteomic analysis of HSC-conditioned media

Aliquots of conditioned media (400 μL each) were used for protein precipitation with 4 volumes of ice-cold acetone. After 2 h incubation at -20°C, samples were centrifuged at 20,000 x g for 10 min. Pellets were air-dried and stored at -20°C until further use.

Combining the lists of proteins identified with gel-free and gel-based secretome analysis resulted in 305 proteins total.

### Gel-free secretome analysis

Protein pellets were dissolved in 0.5 M triethylammonium bicarbonate (TEAB, Sigma Aldrich, St. Louis, MO, USA) and denatured at 60°C for 1 hour. The exact protein concentration was determined employing a Bradford assay, using a serial dilution of bovine serum albumin (BSA, Sigma Aldrich) from 31.25 to 2000 μg/mL in 0.5 M TEAB for calibration. Disulfide bonds were reduced at 60°C for 1 hour by addition of 4.55 mM tris(2-Carboxyethyl)phosphine hydrochloride solution (TCEP-HCl, Sigma Aldrich), followed by alkylation with 8.7 mM iodo acetamide (IAA, Sigma Aldrich) at 24°C for 30 min. Protein digestion was performed overnight at 37°C using trypsin (Promega, Madision, WI, USA) at a ratio of 1:50 to the protein concentration. Digests were dried in a SpeedVac before adjusting peptide concentration to 1 μg/μL in 0.05% trifluoracetic acid (TFA, Sigma Aldrich).

The HPLC instrument was an UltiMate 3000 Nano LC system from Dionex (Germering, Germany) and the mass spectrometer was an LTQ Orbitrap XL from Thermo Scientific (Waltham, MA, USA) equipped with a nano-electrospray ion source. The spray was generated with 10 μm id and 360 μm o.d. fused silica tips from New Objective (Woburn, MA, USA). Tryptic peptides were separated by nano-ion-pair reversed-phase (IP-RP)—HPLC at pH 2.0 on a 150 × 0.20 mm I.D. RP polymer monolith capillary column from Thermo Scientific using a 2-hour gradient of 0–40% acetonitrile in 0.05% aqueous trifluoroacetic acid at a flow-rate of 1 μL/min. The MS1 survey scans of the eluting peptides were executed in the LTQ Orbitrap XL with a resolution of 60,000, recording a window between 450.0 and 2000.0 m/z. The three most intense precursor ions were selected for fragmentation with collision-induced dissociation (CID). The normalized collision energy (NCE) was set at 35.0% for all scans. Data evaluation was performed with Proteome Discoverer (Thermo Scientific) and the open—source library OpenMS.

### Gel-based secretome analysis

Protein pellets were dissolved in 10 μL of LDS-sample buffer and separated on Invitrogen NuPAGE BisTris SDS-gels (4–12%, MOPS-buffer system) with subsequent colloidal Coommassie staining. Lanes were cut into 30 slices of equal size and washed, carbamidomethylated and tryptically digested prior to nano-LC-QTOF-MS/MS analysis as published previously [72]. Tandem mass spectra were searched against the Uniprot database (version 57.15) using the Mascot 2.2 search algorithm (Matrix Science, London, UK) applying the two-peptide-rule.

### IDA modeling

To find HSC gene products that influence gene expression in HCC cells, we applied Intervention-calculus when the DAG (directed acyclic graph) is Absent (IDA) [12]. The algorithm consists of two parts: first, an equivalence class of DAGs is estimated from the observational expression data with the pc-algorithm [13], before causal effects are derived using the graph and intervention calculus [14].

Prior to modeling, gene selection was performed as follows: Gene products secreted from HSC cells were defined as all genes with the terms ‘extracellular’, ‘intercellular’ or ‘secret*’ in any Gene Ontology term or definition. This yielded 1919 genes. Next, genes coding for receptors were removed. The remaining genes were filtered based on expression level, excluding genes that had not been expressed at least in 1/15 CM-stimulated HSC samples at a level larger than the 40th percentile of expression values across all genes and HSC samples. Next, genes with low inter-quartile range, a robust estimate of the variance, across HSC samples were excluded (lowest 20%), yielding 1024 genes annotated to be secreted or present outside of the cell. Next, the overlap between these genes and the gene products detected by mass spectrometry in the HSC-CM (305 gene products) was generated, resulting in 153 gene products. Additionally, growth factors were retained even if they were not detected, as for example IGFs are too small to be monitored by mass spectrometry. This procedure led to a final number of 186 HSC-secreted proteins with a potential influence on HCC cell gene expression going into modeling. The list of HSC secreted gene products is provided in S2 Table.

From the remaining HSC genes, only the genes with highest expression levels (at least 3 samples above the 40th percentile) and with highest inter-quartile range (top 976, such that the total number of HSC genes was 2000) were selected. These genes were supposed to build the network that regulates the secreted genes. On the HCC sample side, genes were selected for differential expression based on significance (q < 0.001), and on log2 fold change (absolute log2 fold change > 1) to focus only on the strongest responses of the HCC cells. This resulted in 227 HCC genes. The filtering procedure is depicted in the left part of Fig 4. Gene expression values were centered and scaled to standard deviation equal to one to make causal effects comparable across genes. From the 2000 HSC genes (secreted and remaining genes), the equivalence class of DAGs was estimated and causal effects were derived from the secreted HSC genes on the selected HCC genes. IDA needs a single tuning parameter, α, which controls the neighborhood size of the graph. It was set to 0.2 as this resulted in the best balance between a not too sparse network and computational burden (higher α values lead to longer running times). To find effects insensitive to small disturbances of the data, IDA was run in a sub-sampling approach adopted from Meinshausen & Bühlmann [73]. For a total of 100 times, 12 out of the 15 samples were drawn, the CPDAG was estimated and causal effects were derived for each DAG in the equivalence class. As a lower bound, the minimum effect of the individual DAGs was retained. The effects were then ranked across all outcome genes (differentially expressed cancer genes) by effect size for each sub-sampling run and the relative frequency of an effect being among the top 30% of effects across all runs was recorded. All effects with a relative frequency equal or above 0.7 were retained for further analysis and the median effect across all sub-samples was recorded. The steps of the causal analysis are schematically shown in the right part of Fig 4.

### Finding the most important regulators

To gain insights into the most important HSC derived regulators of gene expression in HCC, Model-based Gene Set Analysis (MGSA) [24] was employed with the modification that gene sets were redefined as all genes targeted by a specific regulator. For example, the gene set ‘CXCL1’ was comprised of all HCC genes on which CXCL1 exerted a predicted causal effect. MGSA was then used to find a sparse set of regulators explaining the observed differentially expressed genes (q < 0.001, absolute log2 fold change > 1). All predictor-target sets with a posterior probability > b were declared to be the most important regulators. The parameters within MGSA were left at default values, but the size of the gene sets (controlled by the relative frequency cutoff in stability selection) used as input of MGSA was calibrated such that HGF, a known true positive, was in the final list of secreted regulators. While this criterion did not give us unique parameter settings, the remaining genes in the lists resulting from different parameter settings that included HGF were almost identical (S3 Table).

### PAPPA expression in The Cancer Genome Atlas

Un-normalized RNA sequencing and clinical data of liver hepatocellular carcinoma (LIHC) patients was downloaded from The Cancer Genome Atlas (TCGA, http://cancergenome.nih.gov) and normalized using size factors calculated by the R package DESeq2 [74] (function ‘estimateSizeFactorsForMatrix’) and log2-transformed with a pseudo-count of 1 to avoid missing values for samples with zero counts. For the analysis of association of PAPPA expression levels with staging, patients staged with the 7^th^ edition of the AJCC (American Joint Committee on Cancer) that were classified into stages I, II or IIIA were used (n = 199). Stages IIIB, IIIC, IV, and IVA were omitted because of low sample sizes (n<10). For the correlation of PAPPA levels with COL1A levels, all LIHC patients were used (n = 424).

## Supporting Information

S1 Table HSC genes identified based on univariate correlation. Univariate Pearson correlation was calculated between all secreted HSC and CM-responsive HCC genes. HSC genes were ranked based on the number of HCC genes that they correlated with (absolute correlation > 0.7).
(CSV)

S2 Table Secreted proteins used for causal modeling. Proteins detected in at least one of the 5 conditioned media analyzed and annotated growth factors used for causal modeling. Columns hold the following information: ensembl_gene_id: ensembl gene identifier; hgnc_symbol: official gene symbol; uniprot_swissprot: uniprot protein identifier; description: gene description provided by ensembl. Proteins not detected but annotated as growth factors have NA in the protein identifier field.
(CSV)

S3 Table Variation of parameters in causal analysis and identification of secreted HSC regulators. The frequency of an individual effect to be among the top q strongest effects (denoted pi) was varied from 80 to 90 in intervals of 5. Parameter q was varied over the full range of effects in steps of 2% and the median frequency used for selecting stable effects such that for each value of pi, all possible values of q were integrated. Next, MGSA was run on the three lists with different pi values and the median rank over the three MGSA rankings was used for ordering HSC genes. PGF, IGFBP2, PAPPA and HGF are at the top ranks. Values of q and pi outside the range shown did not yield informative lists of targeted HCC genes (either poor coverage or too redundant).
(CSV)

S4 Table PAPPA targets. HCC genes predicted to be regulated by HSC secreted PAPPA. gene_id: ensembl gene identifier; hgnc_symbol: official gene symbol; frequency: frequency of this effect to be among the top 30% strongest effects across sub-sampling runs; median_Effect: median effect size across sub-sampling runs, description: gene description provided by ensembl.
(CSV)

S1 Fig Overrepresented Gene Ontology Biological Process (BP) terms in conditioned media-induced HCC genes. The top 20 terms with smallest Benjamini-Hochberg adjusted p-values are shown.
(PDF)

S2 Fig PAPPA mRNA expression levels in human HSCs and 4 different human HCC cell lines (Hep3B, HepG2, PLC and Huh7). (PDF)

S3 Fig PAPPA protein secretion levels in human HSCs and 4 different human HCC cell lines (Hep3B, HepG2, PLC and Huh7). (PDF)

S4 Fig PAPPA expression correlates with collagen type I expression in HCC tissues from TCGA. (PDF)

S5 Fig PAPPA expression is associated with tumor stage in the TCGA HCC cohort. (PDF)

S6 Fig PAPPA mRNA expression in human HSCs, primary human hepatocytes (PHH) and normal human liver tissues (HLT). (PDF)
