# Supplementary figures and images for: Causal Modeling of Cancer-Stromal Communication Identifies PAPPA as a Novel Stroma-Secreted Factor Activating NFκB Signaling in Hepatocellular Carcinoma
Source: PLoS Comput Biol. 2015 May 28;11(5):e1004293. doi: 10.1371/journal.pcbi.1004293 (PMC4447342; doi:10.1371/journal.pcbi.1004293)

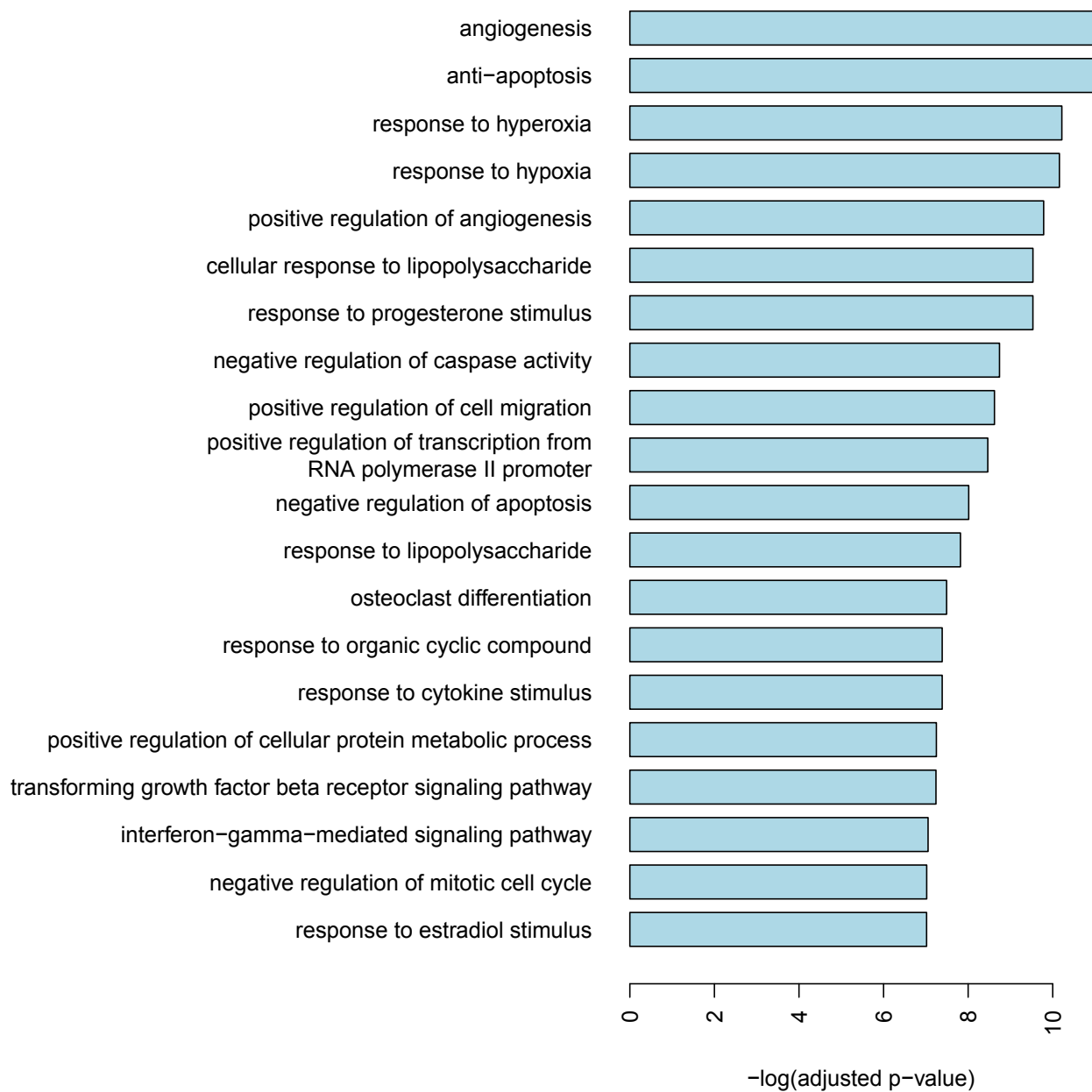

Supplement: S1 Fig — The top 20 terms with smallest Benjamini-Hochberg adjusted p-values are shown. (PDF) [file pcbi.1004293.s005.pdf]

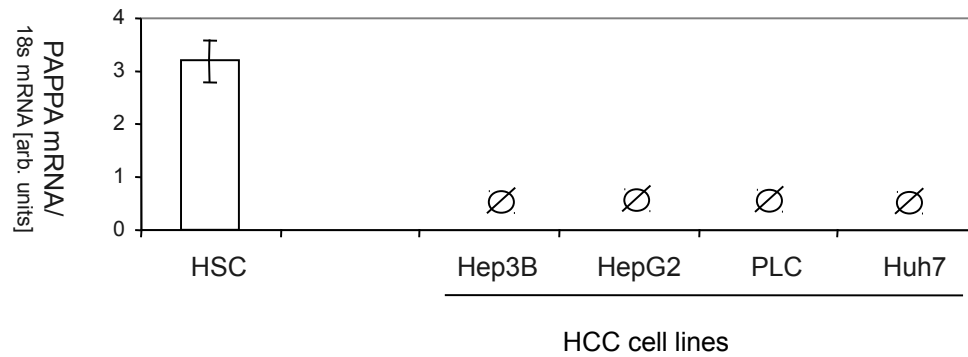

Supplement: S2 Fig — (PDF) [file pcbi.1004293.s006.pdf]

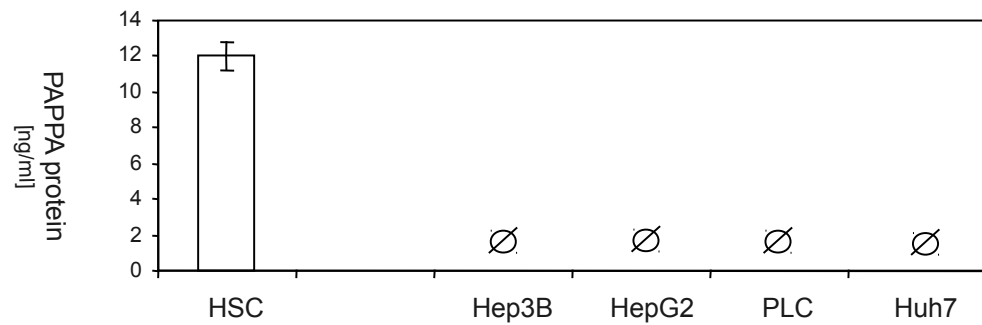

Supplement: S3 Fig — (PDF) [file pcbi.1004293.s007.pdf]

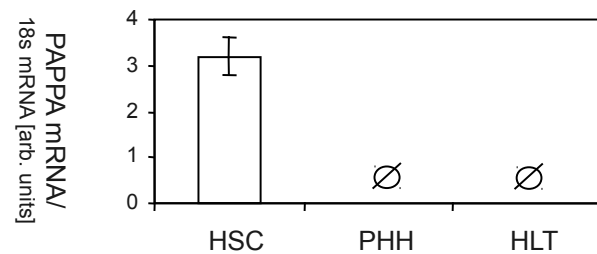

Supplement: S4 Fig — (PDF) [file pcbi.1004293.s008.pdf]

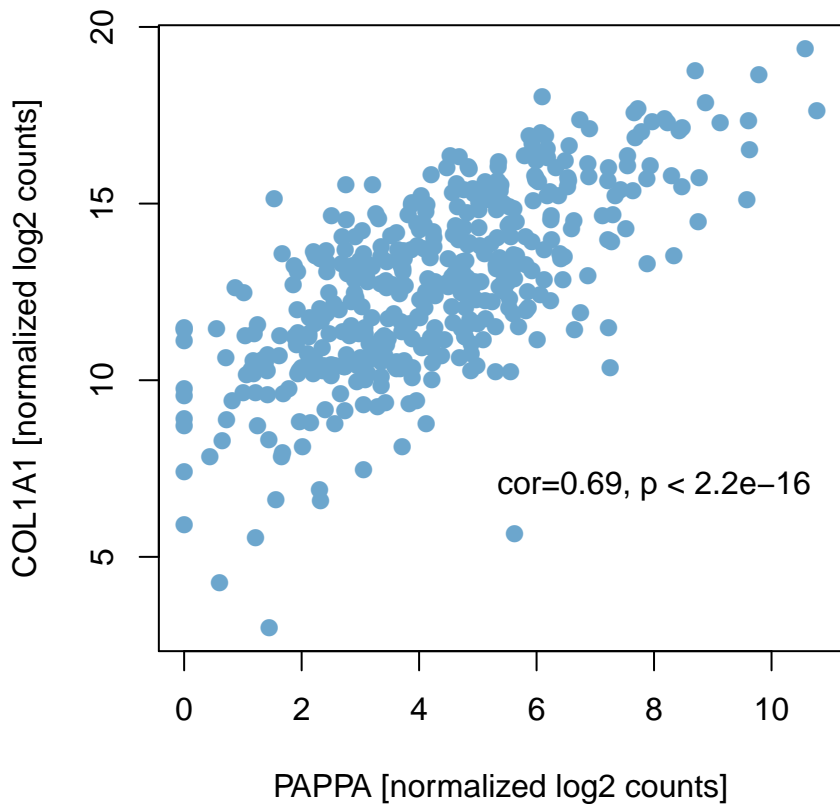

Supplement: S5 Fig — (PDF) [file pcbi.1004293.s009.pdf]

PAPPA [normalized log2 counts]

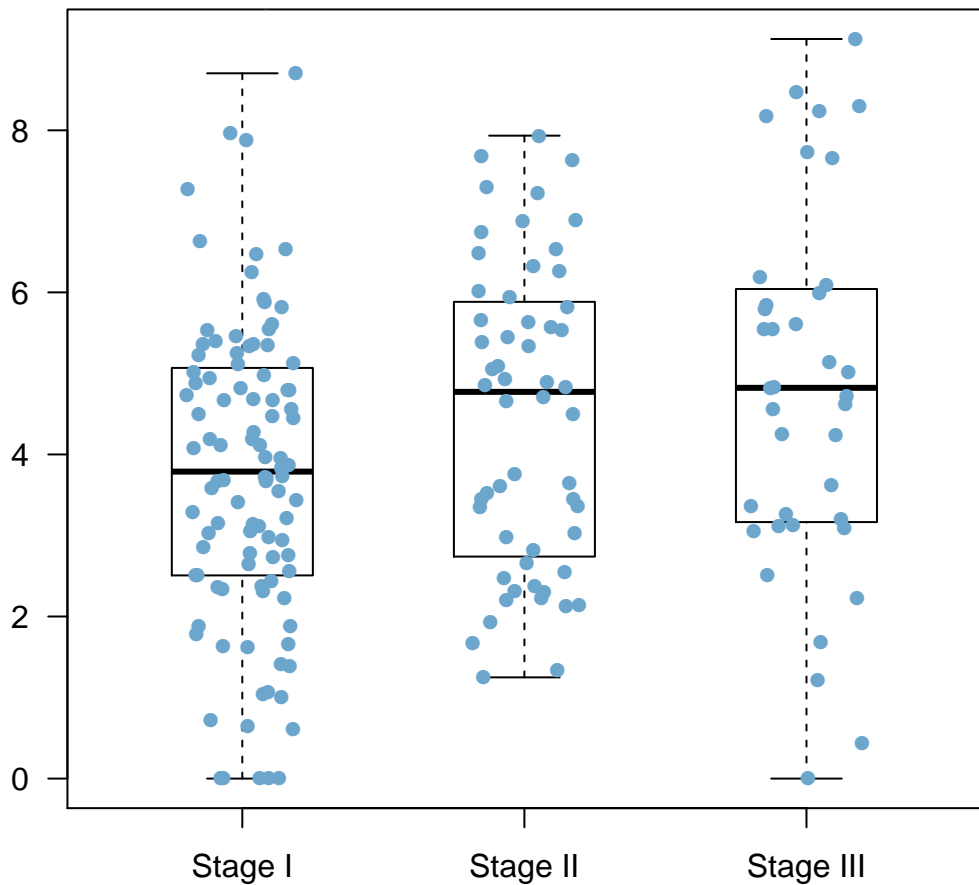

Supplement: S6 Fig — (PDF) [file pcbi.1004293.s010.pdf]
